
JOURNAL INFORMATION
==============================
NLM Title Abbreviation: Behav Neurol
Journal ID: Behav Neurol
NLM Journal ID: 8914585
Journal ID: BN

Behavioural Neurology

ISSN: 0953-4180
EISSN: 1875-8584
Publisher: Wiley

ARTICLE INFORMATION
==============================
PMCID: PMC5469976
PMID: 18430982
DOI: 10.1155/2007/785280
Article ID: 785280
Article version: 1
Subjects: Case Report

Severe Broca’s Aphasia without Broca’s Area Damage

Fridriksson Julius 1 *
Bonilha Leonardo 1 2
Rorden Chris 1
1 Department of Communication Sciences & Disorders University of South Carolina SC USA
2 Department of Neurology Medical University of South Carolina SC USA
*Julius Fridriksson: jfridrik@sc.edu
Print publication date: 2007
Electronic publication date: 2008 Mar 5
Volume: 18
Issue: 4
First page: 237
Last page: 238
Received 2008 Feb 18; Accepted 2008 Feb 18
Copyright: Copyright © 2007 Hindawi Publishing Corporation and the authors.
Copyright year: 2007
License: This is an open access article distributed under the Creative Commons Attribution License, which permits unrestricted use, distribution, and reproduction in any medium, provided the original work is properly cited.
License URL: https://creativecommons.org/licenses/by/3.0/

==============================
Behavioural Neurology 18 (2007) 237–238
IOS Press

Case Report

### Severe Broca's aphasia without Broca's area damage

Julius Fridriksson[a,*], Leonardo Bonilha[a,b] and Chris Rorden[a]
[a]*Department of Communication Sciences & Disorders, University of South Carolina, SC, USA*
[b]*Department of Neurology, Medical University of South Carolina, SC, USA*

Although sub-cortical aphasia is a common neurological disorder, its neural basis is not clear. Recent evidence suggests that hypoperfusion of the cortical language areas may be the primary culprit [1,2]. That is, the subcortical lesion – often including the left striatocapsular region – is associated with decreased cerebral blood flow in the cortex causing impaired language processing. Another account of sub-cortical aphasia suggests that the language impairment is caused by disconnection of the cortical language areas [3]. According to this account, the neurophysiological status of the language areas would be intact but the necessary communication between these areas would be disrupted. Here we present a case of sub-cortical aphasia which would support this latter explanation.

This 32 year old patient suffered a subcortical hemorrhagic stroke during child birth (Fig. 1). Although fMRI showed increased activity in her Broca's area and left middle temporal lobe during an overt picture naming task, it was clear from the tractography that the anterior and posterior language areas were disconnected as white matter tracts originating in Broca's area were not reaching the posterior language areas via the arcuate fasciculus (Fig. 2). This was not the case for the contralateral hemisphere where increased brain activity was noted in the homologues of the anterior and posterior language areas. Although auditory comprehension was relatively intact, this patient presented with severe expressive aphasia. This case demonstrates how a disconnection between the cortical language areas can lead to severely impaired speech production and repetition but relatively preserved language comprehension. Nevertheless, it does not discount sub-cortical aphasia as being the result of impaired cortical hypoperfusion. Rather, this case highlights that different mechanisms may account for impaired language processing in the absence of cortical damage following stroke.

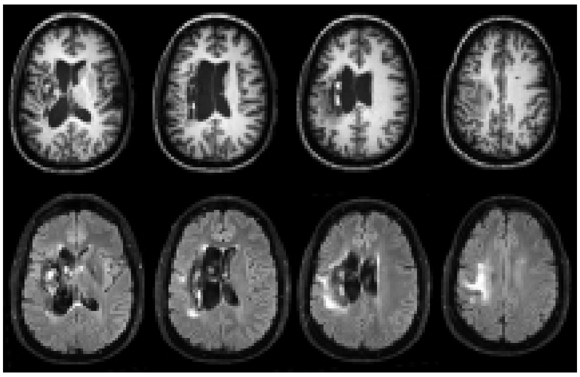

Fig. 1. The stroke involved the left putamen and the arcuate fasciculus resulting in severe Broca's aphasia characterized by the labored production of single words with poor articulation.

### Acknowledgments

This work was supported by grants to JF from the NIDCD (DC008355 & DC005915) and a grant to CR from the NINDS (NS054266).

*Corresponding author: Julius Fridriksson, Ph.D., Associate Professor, Department of Communication Sciences & Disorders, University of South Carolina, SC, USA. Tel.: +1 803 777 4813; Fax: +1 803 777 3081; E-mail: jfridrik@sc.edu.

ISSN 0953-4180/07/$17.00 © 2007 – IOS Press and the authors. All rights reserved

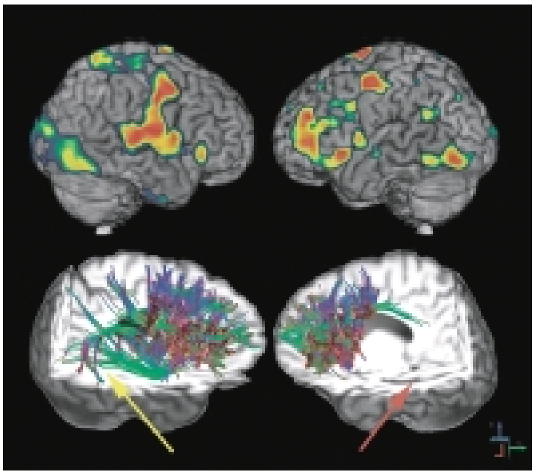

Fig. 2. The fMRI (top) showed increased Broca's and, to a lesser extent, Wernicke's area activity during picture naming attempts. The tractography (bottom) showed where tracts from the right homologue of Broca's area extended via the arcuate fasciculus to the right superior temporal lobe (yellow arrow); this was not the case for the injured left hemisphere (red arrow).

### References

[1] Y.J. Choi, K.H. Lee, D.L. Na, H.S. Byun, S.J. Lee, H. Kim et al., Subcortical aphasia after striatocapsular infarction: Quantitative analysis of brain perfusion SPECT using statistical parametric mapping and a statistical probabilistic anatomic map, *Journ of Nuc Med* **48** (2007), 194–200.

[2] A.E. Hillis, P.B. Barker, R.J. Wityk, E.M. Aldrich, L. Restrepo, E.L. Breese and M. Work, Variability in subcortical aphasia is due to variable sites of cortical hypoperfusion, *Brain and Language* **89** (2004), 524–530.

[3] M.P. Alexander, M.A. Naeser and C.L. Palumbo, Correlations of subcortical CT lesion sites and aphasia profiles, *Brain* **110** (1987), 961–991.
